# Case report: Multiple epstein-barr virus-associated smooth muscle tumours in a child with IL-2-inducible T-cell kinase mutation of undetermined clinical significance

**DOI:** 10.3389/fped.2023.1189219

**Published:** 2023-07-03

**Authors:** Qiongli Wen, Jing Ning, Zhiqun Mao, Xiangdang Long, Xiangling He, Zhihong Chen, Qiuyi Di, Qiaorong Li, Yu Zhuo, Gang Zhong

**Affiliations:** ^1^ Department of Ultrasound, The First Affiliated Hospital of Hunan Normal University/Hunan Provincial People’s Hospital, Changsha, China; ^2^ Department of PET Imaging Center, The First Affiliated Hospital of Hunan Normal University/Hunan Provincial People's Hospital, Changsha, China; ^3^ Department of Pediatric Hematology and Oncology, The First Affiliated Hospital of Hunan Normal University/Hunan Provincial People’s Hospital, Changsha, China; ^4^ Department of Pathology, The First Affiliated Hospital of Hunan Normal University/Hunan Provincial People’s Hospital, Changsha, China

**Keywords:** IL-2-inducible t-cell kinase, mutation, gene, epstein-Barr virus, smooth muscle tumour, immunodeficiency

## Abstract

**Background:**

EBV-associated smooth muscle tumours (EBV-SMTs) are uncommon neoplasms associated with immunodeficiency. The pathogenesis of EBV-SMTs is poorly understood. IL-2-inducible T-cell kinase (ITK), a member of the Tec family of tyrosine kinases, is the predominant Tec kinase in T cells. Researchers have shown that ITK is involved in the pathogenesis of autoimmune diseases and carcinogenesis, and the loss of ITK function due to mutation in patients can lead to EBV-associated lymphoproliferation. Multiple Epstein-Barr virus–associated smooth muscle tumours with ITK mutation have rarely been reported.

**Case presentation:**

A 6-year-old female child was admitted to the hospital due to recurrent bilateral hip pain for more than one year. Tumours were found in the lung, near the intracranial cavernous sinus and in the lumbar spine and paraspinal soft tissues by CT and MRI. The patient underwent vertebral tumour biopsy, which suggested low-grade myogenic or inflammatory myofibroblastic tumours, so the patient was given three courses of chemotherapy without symptom relief or mass reduction. The patient underwent lumbar mass resection, the pathological results indicated EBV-SMT, and a novel germline homozygous deletion mutation in the ITK gene was detected. The deletion mutation in this gene has not yet been reported and the clinical significance of the pathogenicity of the variant is unknown. Intrabronchial mass resection was performed under fibre bronchoscopy, and the pathological results indicated EBV-SMT. No significant recurrence or progression was observed after more than 2 years of follow-up.

**Conclusions:**

We present a rare case of multiple EBV-SMTs combined with ITK gene mutation. Some of the tumours were removed, and some were treated conservatively. There was no significant recurrence or progression after more than two years of follow-up. The optimal treatment regimen still needs to be further explored, and the relationship between ITK gene mutation at this locus and immunodeficiency and EBV-SMT warrants further investigation.

## Introduction

EBV-associated smooth muscle tumours (EBV-SMTs) are uncommon neoplasms that typically manifest in immunodeficient individuals, mostly primary immunodeficient patients, posttransplantation patients and HIV-positive patients (1). Of the three subtypes of EBV-SMTs, the most commonly reported is human immunodeficiency virus–associated smooth muscle tumours (HIV-SMTs), followed by posttransplantation smooth muscle tumours (PT-SMTs), and congenital immunodeficiency disorder–associated smooth muscle tumours (CI-SMTs) have rarely been reported. EBV-SMTs can develop anywhere in the body, and a patient can present with multiple distinct lesions (1). Clinical presentations of EBV-SMTs are related to the site of the tumour and include pain and organ dysfunction (2). IL-2-inducible T-cell kinase (ITK) belongs to the Tec family of kinases and is mainly expressed in T cells. Mutations in the ITK gene are responsible for a variety of diseases. For example, biallelic mutations in the ITK gene locus result in a monogenetic disorder leading to T-cell dysfunction, and ITK deficiency is an immunodeficiency that causes Epstein‒Barr virus (EBV) associated diseases and results in an inability to control EBV infection (3). EBV infection occurs in patients with underlying immunodeficiency and may lead to various lymphoproliferative diseases (LPDs), such as Hodgkin and non-Hodgkin lymphoma, haemophagocytic lymphohistiocytosis (HLH) or smooth muscle tumours (1). The main EBV-SMT therapies are chemotherapy, surgical resection, antiviral therapy, and reduced immunosuppression. Despite the growing number of case reports, the pathophysiology and optimal treatment regimens for EBV-SMTs are not clear. The case we report is regarding a patient who has a novel mutation in the ITK gene. According to the Standards and guidelines for the interpretation of sequence variants: a joint consensus recommendation of the American College of Medical Genetics and Genomics and the Association for Molecular Pathology (4) show that the clinical significance of the pathogenicity of the variant is unknown and the deletion mutation in this gene has not yet been reported, which makes it impossible to confirm the association with immunodeficiency disease and EBV-SMT. Therefore, further explorations and research are still needed.

## Case presentation

The patient, a female aged 6 years, was admitted to our hospital in June 2020.

She presented with recurrent bilateral pain in the hip joints for more than one year, which was worse in the morning and slightly improved in the evening. There was no obvious redness, swelling or fever in either hip joint. Headache manifested 9 days after admission with no accompanying emesis or dysphagia, and limb muscle strength was normal with no ataxia. The patient had a history of hepatitis B but no history of HIV infection. Neurological examination showed normal muscle tone, strength, and reflexes.

**Figure 1:**
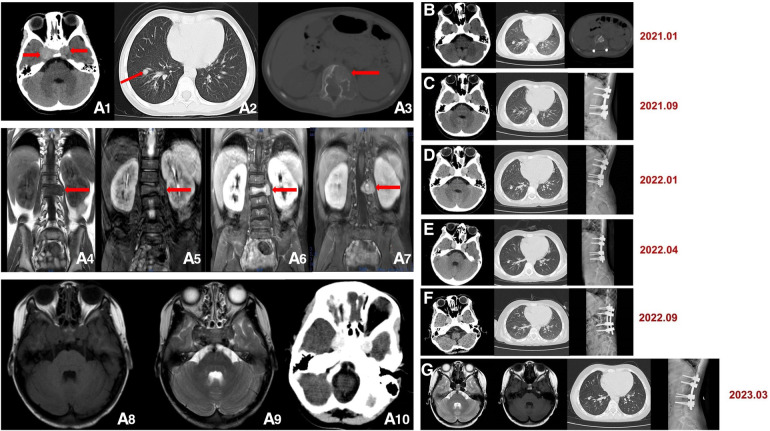
(**A1–10**) Initial computed tomography (CT) and magnetic resonance imaging (MRI) scans. (**A1**) Head CT shows bilateral circular nodules of soft tissue near the cavernous sinus (red arrow), approximately 15 × 13 mm (left) and 20 × 17 mm (right). (**A2**) Lung CT shows multiple nodules in the lungs, with one of the nodules in the right lower lobe and the rest in the basal segment, approximately 7 mm in diameter (red arrow). (**A3**) Lumbar CT shows bone destruction and soft tissue tumours of the L2 vertebral body and left side of the accessory area (red arrow). Lumbar MRI shows an area of hyposignal intensity in (**A4**) T1 imaging and slight hyposignal intensity in (**A5**) T2 imaging (red arrow). (**A6**) and (**A7**) Lumbar-enhanced MRI showed flattening of the L2 vertebral body and bone destruction in the left part of the vertebral body and the vertebral arch adnexa. A 25 × 25 mm local irregular soft tissue lesion was found (red arrow). (**A8**) and (**A9**) Head MRI. (**A10**) Enhanced head CT. (**B–G**) Follow-up images after surgery. Head CT, lung CT and head MRI show that no significant change occurred in the lesions. Lumbar CT and x-ray show postoperative changes.

**Figure 2:**
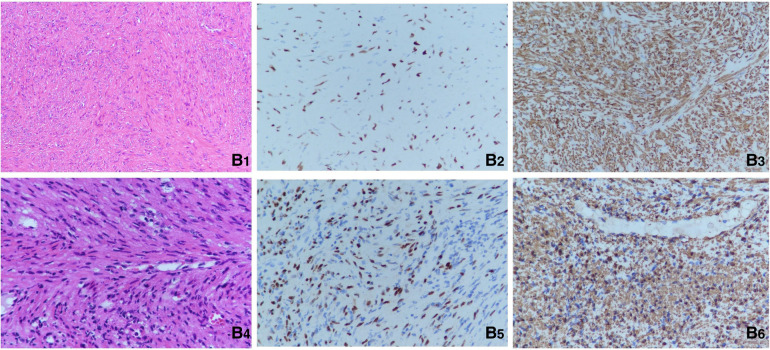
(**B1**) Pathological section of the lumbar spine(hematoxylin and eosinStain, 200×). (**B2**) Positive EBER-ISH indication for the lumbar spine(400×). (**B3**) Immunohistochemistry for smooth muscle actin in the lumbar mass(400×). (**B4**) Pathological section of the right intrabronchial mass(Hematoxylin and EosinStain, 200×). (**B5**) Positive EBER-ISH indication for the bronchial masses(400×). (**B6**) Immunohistochemistry for smooth muscle actin in the bronchial masses(400×).

**Figure 3:**
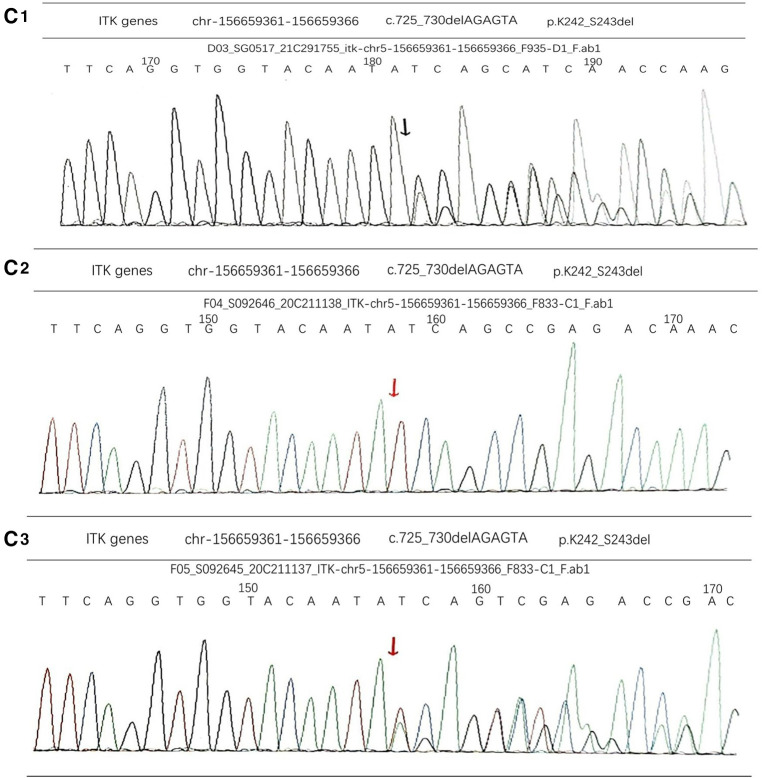
(**C1**) The genetic test results of the father were heterozygous for this mutation. (**C2**) There was a novel mutation in the ITK gene [c.725_730delAGAGTA (p.K242_S243del), located on chromosome 5]. The child was homozygous for this mutation. (**C3**) The genetic test results of the mother were heterozygous for this mutation.

**Figure 4:**
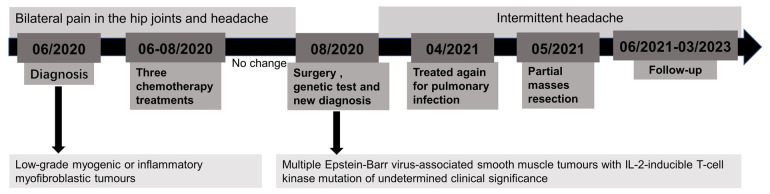
Outline of the patient's treatment record.

The patient underwent the first computed tomography (CT) and magnetic resonance imaging (MRI) examination in our hospital. The CT scans of the head, chest, abdomen and pelvis revealed bilateral nodules of soft tissue near the cavernous sinus; multiple nodules in the lungs, one in the right lower lobe and the rest in the basal segment; and bone destruction and soft tissue tumours of the L2 vertebral body and the left side of the accessory area (Figures 1A1–A3). Lumbar-enhanced MRI showed flattening of the L2 vertebral body and bone destruction in the left part of the vertebral body and the vertebral arch adnexa. A local irregular soft tissue lesion of the lumbar spine with hyposignal intensity in T1 imaging and slight hyposignal intensity in T2 imaging was observed (Figures 1A4,A5). The mass protruded into the spinal canal, and adjacent to the vertebral body, the left psoas major muscle was significantly shifted. However, the boundary with the psoas major muscle was clear. There was mild atrophy and intermediate enhancement of the psoas major muscle on the left side compared to the right side (Figures 1A6,A7). Head MRI revealed bilateral nodular soft tissue signal lesions near the cavernous sinus (Figures 1A8,A9). Head-enhanced CT also showed tumour masses with significant enhancement (Figure 1A10). The patient underwent L2 vertebral tumour biopsy under the guidance of C-Arm x-ray, and the pathological results were spindle cell soft tissue tumours. The cells were arranged in bundles, cell atypia was not obvious, inflammatory cells were rare, and the tumour invaded the vertebra. On the basis of these findings and immunohistochemistry findings, low-grade myogenic or inflammatory myofibroblastic tumours were considered. The patient was given cyclophosphamide, doxorubicin and vincristine (CAV) chemotherapy, considering the malignant transformation and metastasis of the tumour. After three chemotherapy treatments in June, July and August 2020, the symptoms were not relieved, and the masses were not reduced. Considering that the tumours were not sensitive to chemotherapy, it was decided to remove the tumour in the lumbar spine and adnexa area. In August 2020, the patient underwent anterior and posterior lumbar mass resection, fusion bone grafting and internal fixation under general anaesthesia. The patient reported that the symptoms of recurrent bilateral pain in the hip joint had eased after the surgery. Pathological results revealed EBV-SMT in the lumbar spine. The tumour cells were mildly atypical, with no clear mitosis, a low Ki67 proliferation index, no obvious neoplastic necrosis, visible bone destruction, and highly differentiated morphology. In situ hybridization showed EBV-encoded small RNA (EBER) positivity. Immunohistochemistry for smooth muscle actin confirmed the smooth muscle nature of the tumours in the lumbar mass (Figures 2B1–B3). Considering that EBV-SMTs are generally associated with immunodeficiency and EBV infection, we performed EBV antibody, EBV DNA, lymphocyte subset, and genetic tests. With the EB -VCA-lgG>750U/ml(reference:0∼20 U/ml), EB-NA-IgG 48.8U/ml (reference:0∼20 U/ml), the EBV antibody test indicated previous EBV infection. The EBV DNA test detected 6.38 × 10^2^ copies/ml. The lymphocyte subset test results showed the following: CD4 + T% 23.97% (reference: 31.7%∼47%), CD8 + T% 18.3% (reference: 13%∼39%), percentage of total T lymphocytes 49.6% (reference: 63.2%∼77.8%), CD4 + T 516/µl (reference: 646∼1,515/µl), CD8 + T 396/µl (reference: 220∼1,129/µl), and absolute number of total T lymphocytes 1,030/µl (reference: 1,239∼2,611/µl), which suggests the possibility of immunodeficiency disease o·r malignancy, etc. We found a novel mutation in the ITK gene [c.725_730delAGAGTA (p. K242_S243del), located on chromosome 5]; the clinical significance of the pathogenicity of the variant is unknown; and the deletion mutation in this gene has not yet been reported. The child was homozygous for this mutation, and the mother and father were both heterozygous for this mutation (Figure 3). In April 2021, the child was treated again for pulmonary infection, and right intrabronchial mass resection was performed in our hospital under fibre bronchoscopy in May 2021. A pathological analysis of the resected bronchial mass suggested that it was a spindle cell tumour, and on the basis of the pathological findings and medical history, EBV-SMT was confirmed. In situ hybridization detected EBER positivity. Immunohistochemistry for smooth muscle actin confirmed the smooth muscle nature of the tumours in the bronchial masses (Figures 2B4–B6). The EBV DNA test detected 2.5 × 10^2^ copies/ml. There was no recurrence or metastasis in the lumbar tumour and no significant change in the other lesions (Figures 1B–G) more than two years after surgery. The patient had intermittent headaches, and her hip pain disappeared. The patient's follow-up remains ongoing (Figure 4).

## Discussion and conclusions

ITK, a member of the Tec family of tyrosine kinases, is the predominant Tec kinase in T cells. ITK was first reported in the late 1980s (5). ITK is expressed in T lymphocytes and is also expressed in mast cells, natural killer cells and invariant natural killer T cells (iNKT) (1, 6). Numerous studies have shown that ITK is involved in various inflammatory diseases, the pathogenesis of autoimmune diseases and carcinogenesis (7). In the case we report, the patient was found to have a deletion mutation in the ITK gene at c.725_730 delAGAGTA (p. K242_S243del), a mutation at this locus that is not currently reported in the database. Although the mutation was judged to be of no clear clinical significance, the patient presented with T-lymphopenia and multiple EBV-SMTs, and the relationship between mutation at this locus and immunodeficiency and EBV-SMT warrants further investigation.

EBV-SMTs are related to immunosuppression. Three major subtypes of EBV-SMTs are known: CI-SMTs, HIV-SMTs and PT-SMTs (3, 8–10). Although EBV-SMTs are commonly seen in HIV and posttransplantation patients, very few cases of CI-SMTs have been reported. Primary immunodeficiencies associated with SMTs have been reported in the literature, specifically variants of ATM, ADA, IL2RG, ZAP70, GATA2, CARMIL2 and ITK associated with EBV (11). The pathogenesis of these subtypes and the effect of EBV on pathogenesis are poorly understood (8, 9). A study of PT-SMTs showed that they were not associated with EBV infection. Furthermore, some HIV-SMTs were negative for EBV (3, 10). Instead, one hypothesis is that EBV-SMTs are related to the uncontrolled proliferation of EBV-infected lymphocytes and that infected lymphocytes transform smooth muscles via cell proteins produced by the latent membrane protein 1 (LMP-1) viral gene (12–14). Different anatomic sites can be involved in EBV–SMTs, and a patient can contain multiple unique lesions in multiple locations. When multiple tumours are encountered in the same patient, these lesions are considered independent primary lesions rather than metastatic disease (2).

In the majority of case reports on HIV-SMTs to date, the locations most frequently involved were the central nervous system, gastrointestinal tract, skin, liver, larynx, pharynx, and lungs, with tumours rarely occurring in the adrenal glands. In contrast, PT-SMTs occur preferentially in the liver, lungs, pharynx, larynx, spleen, gut, kidneys and brain and rarely in the adrenal gland or iris of the eye. Very few cases of CI-SMTs have been reported, and the most commonly affected sites are the lungs/larynx, followed by the intracranial, liver, adrenal, and splenic soft tissues (9). In our patient, masses were found near the cranial base cavernous sinus, L2 vertebral body, left adnexal area, and lung. Histopathological examinations of the surgically resected masses in the L2 vertebral body, left adnexal area, and lung showed that all were EBV-SMTs.

EBV-SMTs have no specific imaging findings and can be pathologically diagnosed as tumours consisting of well-differentiated fusiform smooth muscle cells and varying numbers of lymphocytes. EBER *in situ* hybridization is the diagnostic test used to confirm the diagnosis of EBV infection and EBV-SMTs, and its performance in immunodeficient patients presenting with spindle-cell proliferation is required (9–15). Among 148 patients evaluated by Hussein et al, EBER *in situ* hybridization showed that 100% of CI-SMTs, 98% of PT-SMTs and 85% of HIV-SMTs were associated with EBV (9).

The main clinical findings of EBV-SMTs are organ dysfunction and pain. Symptoms are mainly associated with the site and size of the tumour (3, 9, 16). For example, intracranial EBV-SMTs are associated with a wide spectrum of symptoms, such as intense and short headaches, aphasia, dizziness, nausea, and darkened vision. Tumour size is an aggravating prognostic factor (16). Our patient presented with recurrent bilateral pain in the hip joints and intermittent headache, which may have been related to lumbar and intracranial lesions. Her hip pain disappeared after surgery.

Treatment strategies are tailored to tumour location, behaviour, and the aetiology of immunosuppression (3, 15). In general, surgical resection, chemotherapy, antiviral therapy and immunosuppression are the mainstays of treatment (15–17). There is no standard treatment for EBV-SMTs due to their intracranial rarity and uncertainty (16).

Allogeneic haematopoietic stem cell transplantation should also be included among the treatment strategies in patients without secondary immunodeficiency disorders. This treatment has been reported to have cured one case of immunodeficiency and EBV + SMTs in a GATA2-deficient patient (11). At the same time, Hernando E et al. discovered a new key role for the AKT-mTOR pathway in smooth muscle transformation and leiomyosarcoma genesis (18), suggesting that it is possible to treat EBV-SMT with targeted drugs. The biological behaviour of EBV-SMTs varies from that of a benign leiomyoma to that of an aggressive fatal tumour unresponsive to intensive chemotherapy, although EBV-SMTs are relatively well-differentiated lesions with only a modest degree of nuclear atypia in histological analyses (19). Total resection of the tumour, a reduction in immunosuppressive therapy and the use of antiretroviral drugs have been reported to result in tumour shrinkage and, in at least one case, complete cure (17–21). Lau KW et al. evaluated 65 patients with EBV-SMTs, the 1-year survival rate was 76.0%, and the 5-year survival rate was 59.6%, indicating a high mortality, even in those with CNS invasion (17). A review of the literature revealed that HIV-SMTs have the poorest prognosis among the three EBV-SMT subtypes. In addition, EBV-SMT patients rarely die from the direct effects of the tumour, and most die from related infectious complications (8).

During the greater than two-year observation of our patient, we found that the tumours were not sensitive to conventional chemotherapy, as after three rounds of chemotherapy, no obvious changes in the lesions were observed. In addition, the size of the nonresected intracranial lesions did not increase throughout the observation period. No recurrence of resected lesions was observed. EBV-SMTs may be indolent and nonfatal. In addition to actively treating the underlying immunodeficiency, surgery might be a good treatment option, and conservative treatment is an option for lesions that are not suitable for resection. The pathophysiology and optimal treatment regimens for congenital immunodeficiency disorder–associated smooth muscle tumours are not clear, and further explorations and research are still needed, and the relationship between ITK gene mutation at this locus and immunodeficiency and EBV-SMT warrants further investigation.

## Data Availability

The original contributions presented in the study are included in the article, further inquiries can be directed to the corresponding author/s.
